# Interaction Potential for NaCs for Ultracold Scattering and Spectroscopy

**DOI:** 10.1021/acs.jpca.2c01810

**Published:** 2022-06-17

**Authors:** Samuel G. H. Brookes, Jeremy M. Hutson

**Affiliations:** Joint Quantum Centre (JQC) Durham-Newcastle, Department of Chemistry, Durham University, South Road, Durham DH1 3LE, United Kingdom

## Abstract

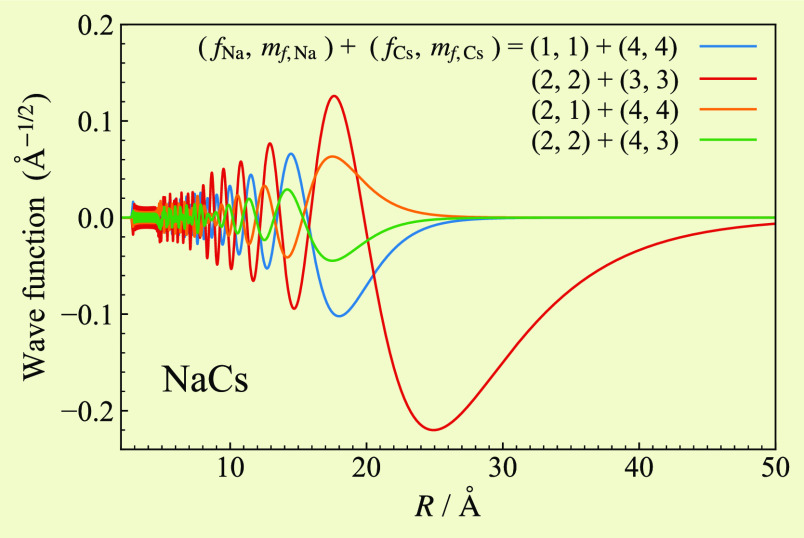

We obtain the interaction potential for NaCs by fitting to experiments on ultracold scattering and spectroscopy in optical tweezers. The central region of the potential has been accurately determined from Fourier transform spectroscopy at higher temperatures, so we focus on adjusting the long-range and short-range parts. We use coupled-channel calculations of binding energies and wave functions to understand the nature of the molecular states observed in ultracold spectroscopy and of the state that causes the Feshbach resonance used to create ultracold NaCs molecules. We elucidate the relationships between the experimental quantities and features of the interaction potential. We establish the combinations of experimental quantities that determine particular features of the potential. We find that the long-range dispersion coefficient *C*_6_ must be increased by about 0.9% to 3256(1)*E*_h_*a*_0_^6^ to fit the experimental results. We use coupled-channel calculations on the final potential to predict bound-state energies and resonance positions.

## 1 Introduction

Ultracold polar molecules have many potential applications, ranging from precision measurement,^1−11^ quantum simulation,^12−17^ and quantum information processing^18−24^ to state-resolved chemistry.^25−30^ A very important class of ultracold molecules are the alkali-metal diatomic molecules; these are usually produced by the association of pairs of ultracold atoms, by magnetoassociation, or by photoassociation, followed by coherent optical transfer to the ground rovibronic state. The ground-state molecules produced in this way include KRb,^31,32^ Cs_2_,^33,34^ Rb_2_,^35^ RbCs,^36,37^ NaK,^38−40^ NaRb,^41^ NaLi,^42^ and NaCs.^43^

A particular success in the past few years has been the production of ultracold NaCs molecules in optical tweezers. Configurable arrays of polar molecules in tweezers offer many possibilities for studying few-body physics involving dipolar species and constructing designer Hamiltonians for quantum logic and quantum simulation. In 2018, Liu et al.^44^ succeeded in loading one atom each of Na and Cs into a single optical tweezer and photoassociated them to form a single electronically excited NaCs molecule in the tweezer. Liu et al.^45^ measured the binding energy of the least-bound triplet state of NaCs by two-photon Raman spectroscopy. Hood et al.^46^ measured interaction shifts for flipping the spin of one or both atoms in the tweezer and located magnetically tunable Feshbach resonances in an excited spin channel. They used these measurements to model the interaction using multichannel quantum defect theory (MQDT). Zhang et al.^47^ located an s-wave Feshbach resonance in the lowest spin channel, allowing them to form a single NaCs molecule in the tweezer by magnetoassociation. Yu et al.^48^ used a different route to form a single NaCs molecule in the tweezer by coherent Raman transfer. Most recently, Cairncross et al.^43^ transferred a molecule formed by magnetoassociation to the absolute ground state by a coherent Raman process.

Studies of ultracold molecule formation typically need close collaboration between experiment and theory. Initial experiments identify properties of the system that can be used to determine an initial interaction potential. The interaction potential is then used to predict new experimental properties. Once these are measured, they are used to refine the interaction potential, and the process repeats. The studies of NaCs in tweezers have followed this cycle several times. In the process, we have learned a considerable amount, both about the specific system and more generally about the ways in which experimental properties are influenced by features of the interaction potential. The purpose of the present article is to present the fitted potential for Na + Cs, describe its relationships to experimental observables, and explain the insights that have been gained. Accurate interaction potentials have applications not only for ultracold molecules but also for precise control of atomic collisions, for example, in studies of Efimov physics^49^ and quantum droplet formation.^50^

The structure of this article is as follows. Section 2 describes the underlying theory and the methods used in the present work. Section 3.1 describes the measured quantities from ultracold scattering and spectroscopy, the wave functions of the underlying weakly bound states, and their relationship to the singlet and triplet potential curves. Section 3.2 describes our procedure for fitting potential parameters, with a focus on how each parameter is related to and constrained by the measured quantities. Section 3.3 describes the near-threshold bound states calculated for our final interaction potential and the resulting scattering properties, including predictions for additional resonances. It compares additional measurements for p-wave and d-wave resonances and gives assignments for the states involved. Finally, Section 4 summarizes our conclusions and the insights gained from the present work.

## 2 Theoretical Methods

### 2.1 Atomic States

The Hamiltonian for an alkali-metal atom *X* in its ground ^2^S state may be written as
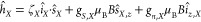
1where ζ_*X*_ is the hyperfine coupling constant, *ŝ*_*X*_ and *î*_*X*_ are the operators for the electron and nuclear spins, respectively, and *ŝ*_*z*,*X*_ and *î*_*z*,*X*_ represent their *z* components along an axis defined by the external magnetic field *B*. We follow the convention of using lowercase letters for operators and quantum numbers of individual atoms and uppercase letters for those of the diatomic molecule or colliding pair of atoms. The constants *g*_*S*,*X*_ and *g*_*n*,*X*_ are the electron and nuclear *g*-factors, and μ_B_ is the Bohr magneton. The numerical values are taken from Steck’s compilations.^51,52^

**Figure 1:**
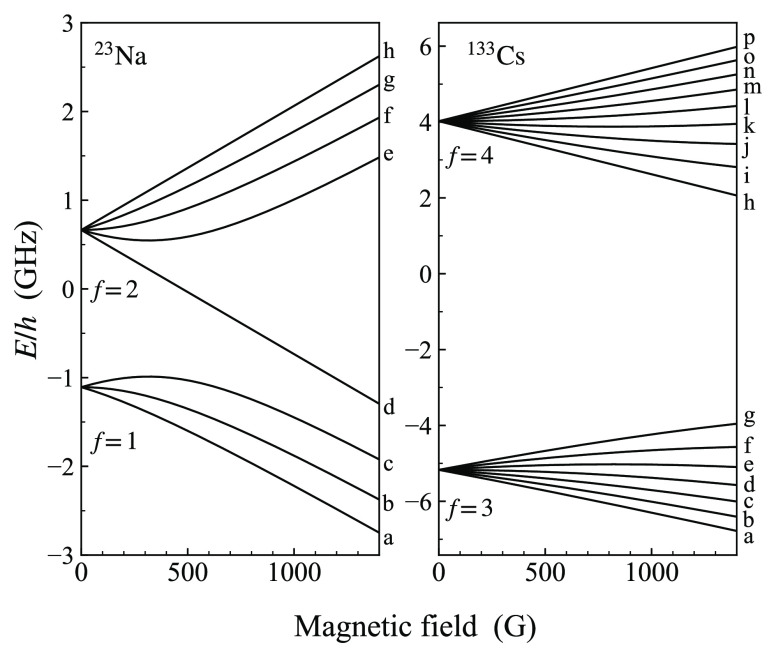
Breit–Rabi plots showing the hyperfine structure and Zeeman splitting for ^23^Na and ^133^Cs atoms. The zero of energy is the hyperfine centroid in each case. Each state is identified by a Roman letter in alphabetic order from the lowest, which is designated as a.

The nuclear spin is *i* = 3/2 for ^23^Na and *i* = 7/2 for ^133^Cs. These are the only stable isotopes for each element, so in the following we omit the mass numbers. The hyperfine splitting at zero field is  and is approximately 1.77 GHz for Na and 9.19 GHz for Cs. Because of these differences, the free atoms have quite different Zeeman structures, as shown in Figure 1.

At low fields, the atomic states may be labeled with  and its projection *m*_*f*_ onto the axis of the magnetic field. However, at higher fields the magnetic field mixes states of different *f* values, particularly for Na. Here we label the states alphabetically in increasing order of energy, with Roman letters from a to h for Na and from a to p for Cs, as shown in Figure 1. In each case, the highest-energy state is spin-stretched, with .

We label a state of an atom pair with two letters, with Na first. For example, ha indicates that Na is in its uppermost state and Cs is in its lowest. The *threshold* for a particular pair state is the energy of the separated atom pair at the appropriate magnetic field. There are 128 = (3 + 5) × (7 + 9) of these thresholds but no more than 16 for a particular value of *M*_*F*_ = *m*_*f*,Na_ + *m*_*f*,Cs_, which is a nearly conserved quantity in a magnetic field.

### 2.2 Two-Atom Hamiltonian

When two alkali-metal atoms in their ground ^2^S states approach one another, their electron spins  couple to form either a singlet state *X*^1^Σ^+^ with total electron spin *S* = 0 or a triplet state *a*^3^Σ^+^ with *S* = 1. Their interaction is governed mostly by the electrostatic potential curves *V*_0_(*R*) and *V*_1_(*R*) for the singlet and triplet states, respectively, but there are also small spin-dependent terms as described below.

The Hamiltonian for an interacting pair of atoms may be written as
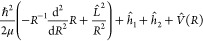
2where *R* is the internuclear distance, μ is the reduced mass, and *L̂* is the operator for the end-over-end angular momentum of the two atoms about one another.

**Figure 2:**
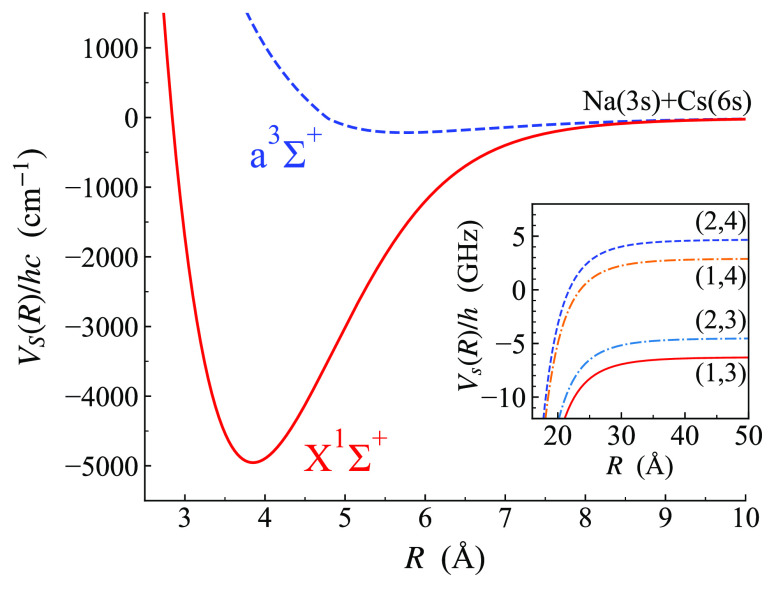
Potential curves of Docenko et al.^53^ for the *X*^1^Σ^+^ and *a*^3^Σ^+^ states of NaCs. The inset shows an expanded view of the zero-field hyperfine structure at long range, with thresholds labeled (*f*_Na_, *f*_Cs_) and energies shown relative to the hyperfine centroid.

The interaction between the atoms is described by the interaction operator, which for a pair of alkali-metal atoms takes the form
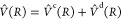
3Here  is an isotropic potential operator that accounts for the potential energy curves *V*_0_(*R*) and *V*_1_(*R*) for the singlet and triplet states. The singlet and triplet projectors  and  project onto subspaces with *S* = 0 and 1, respectively. Figure 2 shows the two potential energy curves for NaCs. The functional forms used for these are described in Section 2.5.

The term *V̂*^d^(*R*) describes the dipole–dipole interaction between the magnetic moments of the electrons at long range, together with terms due to second-order spin–orbit coupling at short range. This makes only small contributions for the experimental observables that we fit to in the present article, but it is important for some of the predicted observables described in Section 3.3. It is described in Appendix A.

### 2.3 Calculations of Bound States and Scattering

We carry out calculations of both bound states and scattering using coupled-channel methods,^54−56^ as described in Appendix B. The total wave function is expanded in a complete basis set of functions for electron and nuclear spins and end-over-end rotation, producing a set of coupled differential equations that are solved by propagation with respect to the internuclear distance *R*. The coupled equations are identical for bound states and scattering, but the boundary conditions are different.

Scattering calculations are performed with the MOLSCAT package.^57,58^ Such calculations produce the scattering matrix **S** for a single value of the collision energy and magnetic field each time. The complex s-wave scattering length *a*(*k*_0_) is obtained from the diagonal element of **S** in the incoming channel, *S*_00_, using the identity^59^
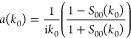
4where *k*_0_ is the incoming wavenumber related to the collision energy *E*_coll_ by *E*_coll_ = *ℏ*^2^*k*_0_^2^/(2μ). The scattering length *a*(*k*_0_) becomes constant at sufficiently low *E*_coll_, with limiting value *a*. In the present work, s-wave scattering lengths are calculated at *E*_coll_/*k*_B_ = 1 nK, which is low enough to neglect the dependence on *k*_0_.

A zero-energy Feshbach resonance occurs where a bound state of the atomic pair (diatomic molecule) crosses a scattering threshold as a function of the applied field. At the lowest threshold, or in the absence of inelastic processes, the scattering length is real. Near a resonance, *a*(*B*) passes through a pole and is approximately
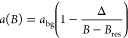
5where *B*_res_ is the position of the resonance, Δ is its width, and *a*_bg_ is a slowly varying background scattering length. In the presence of inelastic processes, *a*(*B*) is complex and the pole is replaced by an oscillation.^59^ MOLSCAT can converge on Feshbach resonances automatically and characterize them to obtain *B*_res_, Δ, and *a*_bg_ (and the additional parameters needed in the presence of inelasticity) as described in ref (60).

Coupled-channel bound-state calculations are performed using the packages BOUND and FIELD,^58,61^ which converge upon bound-state energies at fixed field and upon bound-state fields at fixed energy, respectively. The methods used are described in ref (62). Once bound states have been located, their wave functions may be obtained by back-substitution using matrices saved from the original propagation.^63^ Alternatively, the expectation value of any operator may be calculated by finite differences, without requiring explicit wave functions.^64^ This capability is used here to calculate overall triplet fractions for bound states.

Zero-energy Feshbach resonances can be fully characterized using MOLSCAT as described above. However, if only the position of the resonance is needed, it is more convenient simply to run FIELD at the threshold energy to locate the magnetic field where the bound state crosses the threshold.

A key capability of both MOLSCAT and FIELD, used in the present work, is automated convergence of any one parameter in the interaction potential to reproduce a single observable quantity, such as a bound-state energy, scattering length, or resonance position. This uses the same algorithms as are used to converge on such quantities as a function of the external field.^60,62^

In the present work, the coupled equations for both scattering and bound-state calculations are solved using the fixed-step symplectic log-derivative propagator of Manolopoulos and Gray^65^ from *R*_min_ = 4*a*_0_ to *R*_mid_ = 30*a*_0_, with an interval size of 0.002*a*_0_, and the variable-step Airy propagator of Alexander and Manolopoulos^66^ between *R*_mid_ and *R*_max_ = 10 000*a*_0_. The exception to this is calculations used to plot wave functions, which use the fixed-step log-derivative propagator of Manolopoulos.^63,67^

### 2.4 Basis Sets for Angular Momentum

To carry out coupled-channel calculations, we need a basis set that spans the space of electron and nuclear spins and of relative rotation. We do not require a basis set where the atomic Hamiltonians *ĥ*_1_ and *ĥ*_2_ are diagonal because MOLSCAT transforms the solutions of the coupled equations into an asymptotically diagonal basis set before applying scattering boundary conditions.

There are five sources of angular momentum for an interacting pair of alkali-metal atoms: the electron spins *s*_1_ and *s*_2_, the nuclear spins *i*_1_ and *i*_2_, and the rotational angular momentum *L*. These may be coupled together in several different ways, and different coupling schemes are useful when discussing different aspects of the problem. The separated atoms are conveniently represented by quantum numbers (*s*, *i*)*f*, *m*_*f*_, where the notation (*a*, *b*)*c* indicates that *c* is the resultant of *a* and *b* and *m*_*c*_ is the projection of *c* onto the *z* axis. Conversely, the molecule at short range (and low field) is better represented by *S* and the total nuclear spin *I*, together with their resultant *F* and its projection *M*_*F*_. In the present work, we carry out coupled-channel calculations in two different basis sets. The first is
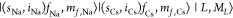
6which we term the coupled-atom basis set. The second is
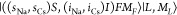
7which we term the *SIF* basis set. The only conserved quantities in a magnetic field are *M*_tot_ = *m*_*f*,Na_ + *m*_*f*,Cs_ + *M*_*L*_ = *M*_*F*_ + *M*_*L*_ and parity (−1)^*L*^. We take advantage of this to perform calculations for each *M*_tot_ and parity separately. In each calculation, we include all basis functions of the required *M*_tot_ and parity for , , and , subject to the limitation *L* ≤ *L*_max_. In most of the calculations in the present work, *L*_max_ = 0, except that we use *L*_max_ = 1 for calculations of p-wave states and resonances in Section 3.3.4 and *L*_max_ = 2 for the calculations in Section 3.3.3.

### 2.5 Singlet and Triplet Potential Curves

Our starting points for fitting the interaction potentials are the singlet and triplet potential curves of Docenko et al.,^53^ shown in Figure 2. These were fitted to extensive Fourier transform (FT) spectra involving vibrational levels of up to *v* = 83 in the singlet state, which has a total of 88 levels, and of up to *v* = 21 in the triplet, which has 25. These curves give an excellent representation of the levels they were fitted to, but their behavior at higher energies depends sensitively on how they are extrapolated, and they do not reproduce the near-threshold states important for ultracold scattering.

In a central region from *R*_SR,*S*_ to *R*_LR,*S*_, with *S* = 0 for the singlet and *S* = 1 for the triplet, each curve is represented as a finite power series in a nonlinear function ξ_*S*_ that depends on the internuclear separation *R*,
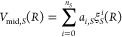
8where
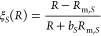
9The quantities *a*_*i*,*S*_ and *b*_*S*_ are fitting parameters, and *R*_m,*S*_ is chosen to be near the equilibrium distance for the state concerned. The values of the parameters fitted to FT spectroscopy for NaCs are given in Tables 1 and 2 of ref (53); the values *R*_SR,0_ = 2.8435 Å and *R*_SR,1_ = 4.780 Å, which specify the minimum distance at which the power-series expansion is used for each state, are particularly important for the present work.

At long range (*R* > *R*_LR,*S*_), the potentials are
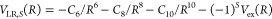
10where the dispersion coefficients *C*_*n*_ are common to both potentials. The long-range matching points are chosen as *R*_LR,0_ = *R*_LR,1_ = 10.2 Å. The exchange contribution is^68^
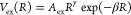
11where *a*_0_ is the Bohr radius. It makes an attractive contribution for the singlet and a repulsive contribution for the triplet. The value of *C*_6_ used by Docenko et al.^53^ was fixed at the theoretical value of Derevianko et al.,^69^ whereas *C*_8_, *C*_10_, and *A*_ex_ were fitting parameters. The mid-range potentials are adjusted to match the long-range potentials at *R*_LR,*S*_ by setting the constant terms *a*_0,*S*_ in eq 8 as required.

Finally, the potentials are extended to short range (*R* < *R*_SR,*S*_) with simple repulsive terms,
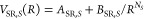
12where *A*_SR,*S*_ is chosen so that *V*_SR,*S*_ and *V*_mid,*S*_ match at *R*_SR,*S*_. In the present work, *B*_SR,*S*_ is chosen to match the derivative of these two functions. However, this latter constraint was not applied in ref (53), producing discontinuities in the derivatives of the potential curves at *R*_SR,*S*_.

## 3 Results and Discussion

### 3.1 Observables from Ultracold Scattering and Spectroscopy

The recent experimental studies on Na + Cs in tweezers^43,45−48^ have measured a number of quantities that could be used in fitting potential curves. Each observable is associated with one or more molecular bound states of a particular spin character. In this section, we consider each observable quantity and the nature of the corresponding state in order to understand how the observable depends on features of the singlet and triplet potential curves. The calculations in this section are based on “lightly fitted” potential curves, with approximately correct scattering lengths. Calculations based on the final potential would be visually almost identical.

#### 3.1.1 General Features of Near-Threshold States

The near-threshold states that are important in studies of ultracold molecules and ultracold collisions are typically bound by less than a few GHz. Their wave functions extend several nm to distances where hyperfine coupling is stronger that the spacing between the singlet and triplet curves. This long-range region is shown as an inset in Figure 2. Each curve represents a different zero-field hyperfine threshold, labeled (*f*_Na_, *f*_Cs_). For an interaction potential of the form −*C*_6_/*R*^6^ at long range, the bound states below each threshold are located within “bins” given by multiples of an energy scale *E̅* = *ℏ*^2^/(2*μa̅*^2^),^70^ where *a̅* is the mean scattering length^71^ and depends only on *C*_6_ and μ. For NaCs, *a̅* = 59.17*a*_0_ and *E̅* = 26.30 MHz. The first (top) bin is 36.1*E̅* = 950 MHz deep, implying that the top (least-bound) bound state for each spin combination lies 0 to 950 MHz below its threshold; the position of the state within the bin is governed by the actual scattering length *a*, which differs for different thresholds. The least-bound state is designated *n* = −1. The second and third bins extend to depths of 249*E̅* and 796*E̅*, so the second and third bound states (with *n* = −2 and −3) lie between 950 MHz and 6.6 GHz and between 6.6 and 21 GHz below the threshold, respectively. We focus here on states with binding energies within the three uppermost bins; accurately modeling this region of the potential is crucial for obtaining reliable scattering lengths and resonance positions, among other properties.

#### 3.1.2 Binding Energy of the Absolute Ground State

Cairncoss et al.^43^ have measured the energy of the absolute ground state of NaCs, initially with respect to the near-threshold state formed by magnetoassociation. After correcting for hyperfine and Zeeman effects and the binding energy of the near-threshold state, they infer that the binding energy *E*_00_ of the lowest rovibrational level of the singlet state, relative to the hyperfine centroid of free atoms, is 147 044.63(11) GHz.

This state is located thousands of cm^–1^ below the minimum of the triplet state, so singlet–triplet mixing is negligible. Its binding energy is sensitive only to the singlet curve. Its wave function is tightly confined around the minimum of the singlet curve near 3.85 Å, and the zero-point energy is very well determined by the FT spectra, so it is mostly sensitive to the well depth of the singlet curve.

#### 3.1.3 Binding Energy of the Least-Bound Pure Triplet State

The binding energy of the least-bound state in the hp channel, *E*_–1_^hp^, has been measured by Liu et al.^45^ and refined by Hood et al.^46^ This channel corresponds to (*f*, *m*_*f*_) = (2, 2) for Na and (4, 4) for Cs. Both of these states are spin-stretched, with *f* = *m*_*f*_ = *s* + *i*, so states that lie in the hp channel are pure triplet in character. The binding energy of the state, relative to the hp threshold, is 297.6(1) MHz at 8.8 G.

The binding energy *E*_–1_^hp^ is sensitive only to the triplet curve. It is also very closely related to the triplet scattering length *a*_t_, with only slight sensitivity to the dispersion coefficient *C*_6_ and even less to *C*_8_ and *C*_10_.

#### 3.1.4 Binding Energy of the Least-Bound State in the ha Channel

Yu et al.^48^ have measured the binding energy of the least-bound state in the ha channel, *E*_–1_^ha^, with respect to the ha threshold. The binding energy is 770.1969(2) MHz at *B* = 8.83 G.

**Figure 3:**
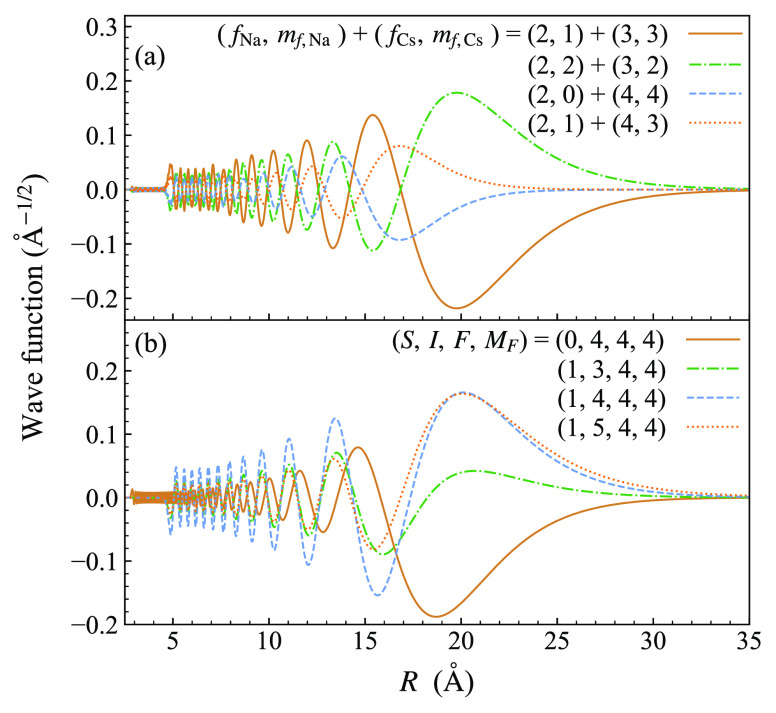
Components of the wave function for the least-bound state in the ha channel, shown in both the (a) coupled-atom and (b) *SIF* representations. Components in all four contributing channels are plotted in each case.

The ha channel corresponds to (*f*, *m*_*f*_) = (2, 2) for Na and (3, 3) for Cs, so *M*_*F*_ = 5. Because there are four atomic pair states with *M*_*F*_ = 5, which are mixed by the interaction potential, this state has a mixture of singlet and triplet character. To quantify this, Figure 3 shows the components of the wave function for this state. In the coupled-atom representation, the main contribution is provided by the ha channel, with smaller contributions arising from the other three channels with *M*_*F*_ = 5. In the *SIF* representation, there are similar contributions from singlet and triplet channels. The overall triplet fraction obtained from the expectation value of the triplet projector  is 49.7%.

The binding energy *E*_–1_^ha^ is approximately equally sensitive to the singlet and triplet curves. It is closely related to the scattering length in the ha channel. However, because the triplet scattering length is determined independently by *E*_–1_^hp^, the role of *E*_–1_^ha^ is to provide information on the singlet scattering length *a*_s_.

#### 3.1.5 Position of Feshbach Resonance in the aa Channel

Zhang et al.^47^ have observed a strong s-wave resonance in the lowest hyperfine channel at 864.11(5) G and used it to form NaCs molecules by magnetoassociation. The atoms collide at the aa threshold, corresponding to (*f*, *m*_*f*_) = (1, 1) + (3, 3) at low field. The resonance position is designated *B*_res_^aa^.

**Figure 4:**
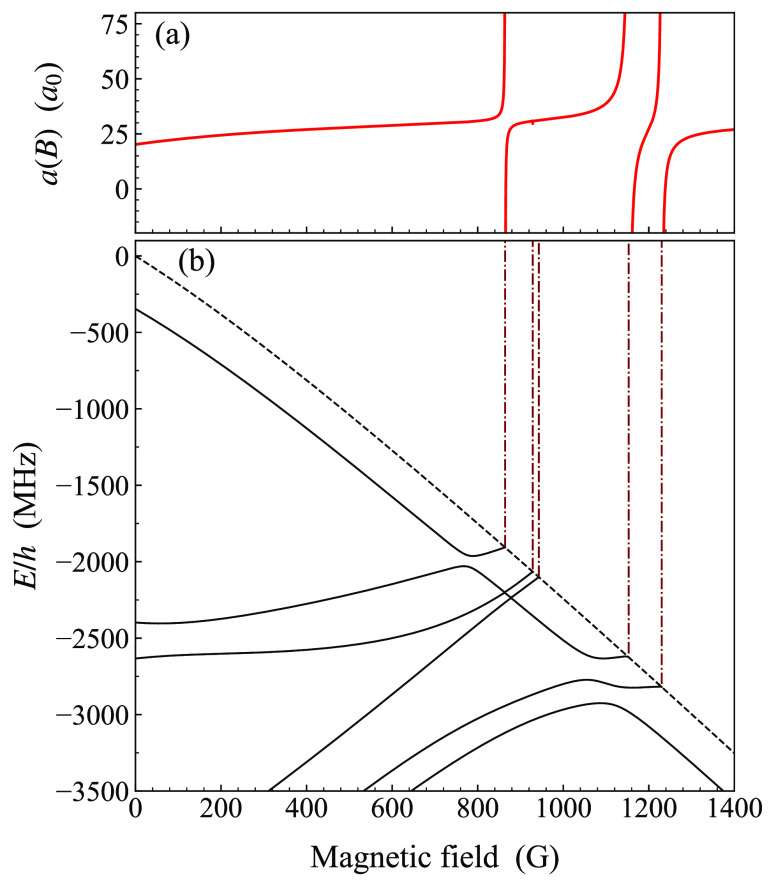
(a) Calculated s-wave scattering length in the aa channel as a function of magnetic field. (b) Energies of weakly bound s-wave molecular states with *M*_*F*_ = 4 (solid lines) and of the aa threshold (dashed line). The zero of energy is the zero-field threshold energy. Feshbach resonances occur where bound states cross the threshold and are indicated by vertical lines extending up to the corresponding position on the plot of the scattering length.

Figure 4 shows the pattern of s-wave bound states below the aa threshold as a function of magnetic field, obtained from coupled-channel bound-state calculations, together with the calculated scattering length. The bound state originating at −400 MHz and running parallel to the aa threshold has the same spin character (i.e., the same spin quantum numbers) as the aa threshold. The resonance near 864 G occurs when this state is pushed up and across the threshold by a more deeply bound state through an avoided crossing.

**Figure 5:**
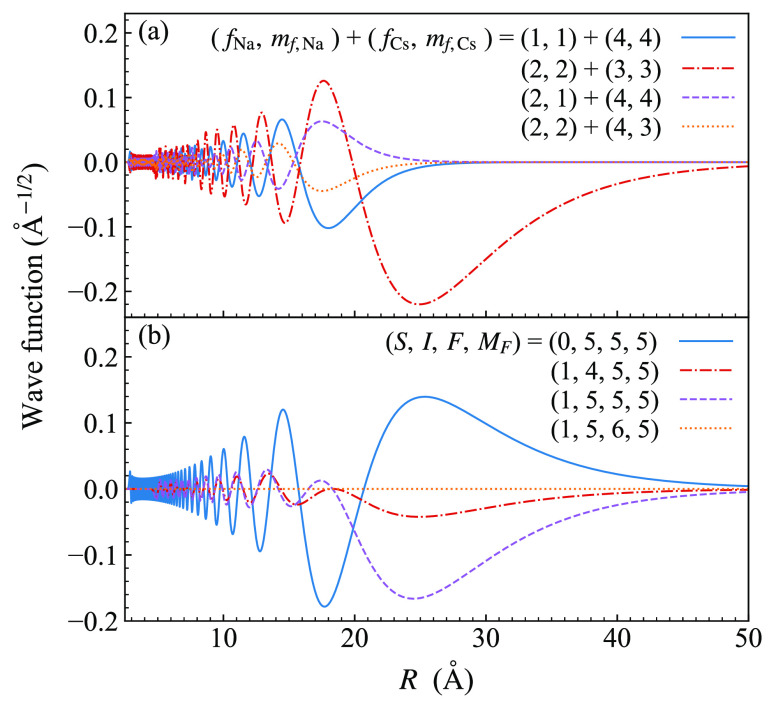
Components of the wave function at zero field for the state responsible for the resonance near 864 G in the aa channel, shown in both the (a) coupled-atom and (b) *SIF* representations. Components in the four most prominent channels are plotted in each case.

The more deeply bound state originates from −2450 MHz below the aa threshold at zero field. Its depth and behavior with magnetic field ultimately determine the location and nature of the resulting resonance. The components of its wave function at zero field are plotted in Figure 5. In the coupled-atom representation, the dominant components are from channels corresponding to (*f*_Na_, *f*_Cs_) = (2, 3) (solid brown and dotted–dashed green curves). The calculated zero-field binding energy is 4220 MHz below the (2, 3) threshold, indicating that the state corresponds to *n* = −2. Because of this, the wave function is concentrated at significantly shorter range than those for the least-bound states in Figure 3. The components of the wave function in the *SIF* representation are shown in Figure 5(b). There are significant contributions from both singlet and triplet channels. The overall triplet fraction is 69.5%.

#### 3.1.6 Position of Feshbach Resonance in the cg Channel

Hood et al.^46^ have measured the position of an inelastic loss feature in the cg channel at 652.1(4) G. This channel corresponds to (*f*, *m*_*f*_) = (1, −1) + (3, −3) at low field. They attributed this feature to an s-wave Feshbach resonance, and its position is designated *B*_res_^cg^.

**Figure 6:**
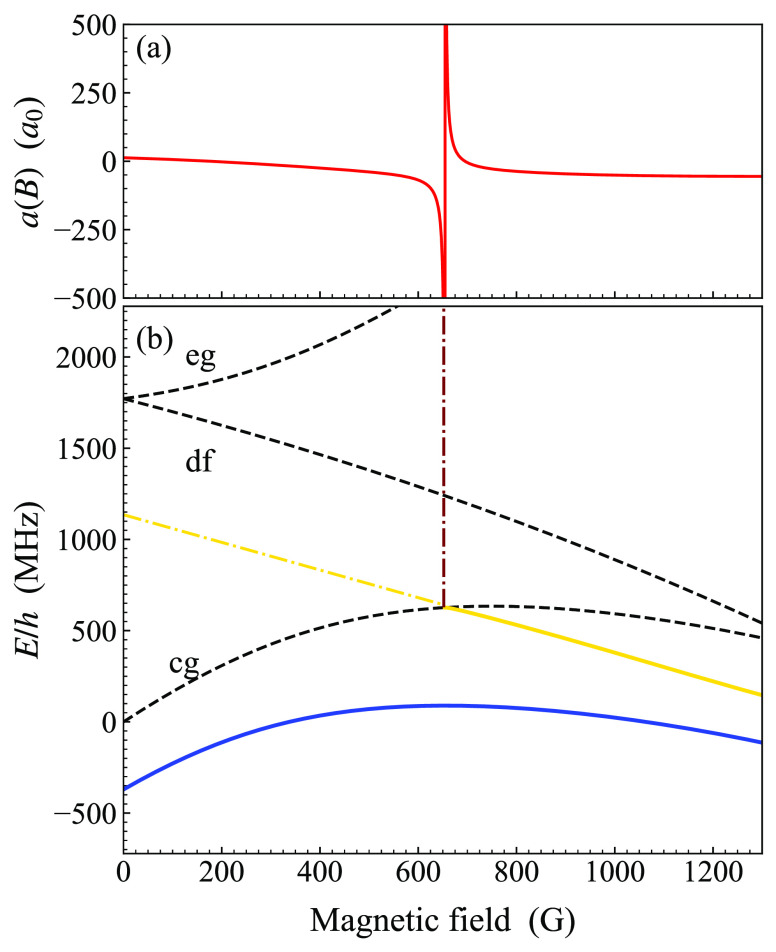
(a) Calculated s-wave scattering length in the cg channel as a function of magnetic field. (b) Energies of weakly bound s-wave molecular states with *M*_*F*_ = −4 (solid lines) and of nearby thresholds (dashed lines). The zero of energy is the zero-field energy of the cg and aa thresholds. The resonant state (yellow) is approximately parallel to the df threshold, and there is another state (blue) roughly parallel to the cg threshold. The resonance position is marked by a vertical line extending up toward the scattering-length plot. The dotted–dashed yellow line shows a linear extrapolation of the resonant state to zero field.

The state that causes this resonance crosses downward across the threshold with increasing magnetic field. It is bound at fields above the crossing but is quasibound at fields below it, so it cannot as simply be traced back to its origin at zero field with BOUND. Figure 6 shows the bound states and atomic thresholds with *M*_*F*_ = −4 relevant to this resonance. A least-squares fit to the crossing state (solid yellow line) at fields above the crossing gives a gradient of −0.76 MHz/G and a zero-field intercept of −5140 MHz. The state is reasonably parallel to the df threshold with (*f*, *m*_*f*_) = (2, −2) + (3, −2), which has a gradient of about −0.7 MHz/G; we conclude that the state is mostly of df character. Calculation of the wave function at a field 80 G above the crossing confirms this, though there is developing coupling to the state in the cg channel (solid blue line) with increasing field. The state is bound by about 640 MHz with respect to the df threshold, indicating that it lies in the top bin. Its overall triplet fraction is 60.6%.

This state has a roughly similar triplet fraction and binding energy (with respect to the threshold that supports it) as the least-bound state in the ha channel. However, the interpretation of the position of the loss peak is somewhat uncertain. First, the resonance is quite broad, as seen in Figure 6(a), with width Δ of around 40 G. Secondly, Brooks et al.^72^ have shown that inelastic loss features for atom pairs in tweezers may be significantly shifted from the actual resonance position. We therefore conclude that the information on the interaction potential available from this feature is similar to but less reliable than that available from *E*_–1_^ha^; we therefore do not use *B*_res_^cg^ in fitting.

#### 3.1.7 Interaction Shifts and Derived Scattering Lengths

Hood et al.^46^ have measured interaction shifts for spin-flip transitions of Na atoms (transition a ↔h) and Cs atoms (transition a↔p) in tweezers. The shifts are defined as the difference in transition frequency between a tweezer containing one atom of each species and a tweezer containing a single atom. They are made up of shifts for individual pair states that depend on the scattering length for the particular pair of atomic states. However, modeling the shift for two different atoms in a nonspherical tweezer involves a complicated forward calculation to take account of the anisotropy of the trap and the coupling between the relative and center-of-mass motions of the atoms.^46^

Hood et al. used their measurement of *E*_–1_^hp^ to extract a triplet scattering length *a*_t_ = 30.4(6)*a*_0_. They used this to calculate the interaction shift for the hp state of Na + Cs and hence to extract interaction shifts for the ha and ap states from the transition frequencies. They found an interaction shift of −30.7 kHz for the ha state, from which they inferred a large negative scattering length of −693.8*a*_0_. From this, they used MQDT to extract a singlet scattering length *a*_s_ = 428(9)*a*_0_.

The measurements of interaction shifts are principally sensitive to the scattering length for the ha state. They contain information that is very similar to *E*_–1_^ha^ but is less precise and far less direct. We therefore do not use them in fitting.

### 3.2 Fitting Potential Parameters

The interaction potentials of Docenko et al.^53^ were fitted primarily to FT spectra, which accurately determine the deeper part of the potential but not the near-threshold part. Our goal is to adjust the potential curves to fit the ultracold observables described above while retaining as much as possible their ability to reproduce the FT spectra. We therefore keep the two power series that represent the singlet and triplet potential wells fixed, with the coefficients obtained in ref (53), and vary only the short-range and long-range extrapolations. As will be seen below, we found it necessary to make small changes in the long-range dispersion coefficients *C*_6_ and *C*_8_ of eq 10 as well as to vary the parameters of the short-range extrapolations, *R*_SR,*S*_ and *N*_*S*_ of eq 12.

There is no advantage in varying *R*_LR,*S*_, the point at which the mid-range power series (eq 8) is matched to the long-range exchange-dispersion potential (eq 10). As described above, continuity of the curves at *R*_LR,*S*_ is achieved by shifting the midrange curves bodily using the constant terms *a*_0,*S*_ in the power series. Any change in the dispersion coefficients *C*_6_ and *C*_8_ thus shifts the minima of both curves and is directly reflected in the binding energy *E*_00_ of the absolute ground state. The measured value of *E*_00_ effectively provides a constraint that relates *C*_8_ to *C*_6_.

For a single potential curve *V*(*R*) that varies as −*C*_6_/*R*^6^ at long range, the scattering length *a* is approximately related to a phase integral Φ by^71^
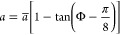
13where
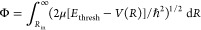
14and *R*_in_ is the inner classical turning point at the threshold energy *E*_thresh_. With the mid-range and long-range parts of the curve fixed by other observables, the only way to adjust *a* is to vary the short-range potential in the region between *R*_in_ and *R*_SR_, where it is given by eq 12. Because the relationship between *a* and the binding energy *E*_–1_ is only very weakly affected by the dispersion coefficients, the same applies to *E*_–1_. These considerations apply independently to the singlet and triplet curves, so we have dropped the *S* subscript here.

If *A*_SR_ and *B*_SR_ are chosen to give continuity of the potential and its derivative at *R*_SR_, then the short-range extrapolation (eq 12) for each curve has free parameters *R*_SR_ and *N*. The short-range power *N* controls the hardness of the repulsive wall and can substantially affect the extrapolation of the potential to energies above dissociation, which are important for higher-energy collisions. Nevertheless, in potentials fitted to FT spectra, *N* has commonly been assigned an arbitrary fixed value, which has ranged from 3 for NaCs^53^ to 12 for K_2_.^73^ A requirement to reproduce a particular value of *a* or *E*_–1_ is satisfied along a line in the space of *R*_SR_ and *N*. However, because of the longer-range contribution to the phase integral Φ, this line depends significantly on the values of *C*_6_ and *C*_8_.

**Figure 7:**
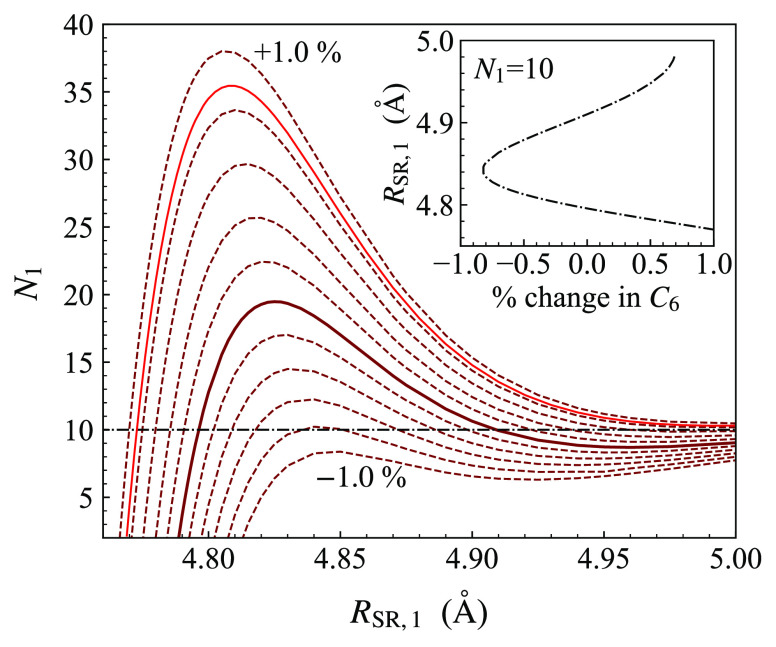
Relationship between the inverse power *N*_1_ and the short-range matching point *R*_SR,1_ required to reproduce the experimental binding energy *E*_–1_^hp^ of the least-bound triplet state of NaCs. The relationship is given for various values of the dispersion coefficient *C*_6_, expressed as percentage differences from the theoretical value.^69^ The solid brown line shows the value used in ref (53), and the solid red line shows the final value of the present work. The inset shows the dependence of *R*_SR,1_ on *C*_6_ for the choice *N*_1_ = 10.

We apply this approach first to the potential curve for the triplet state. As described above, the FIELD package can automatically converge on the value of a potential parameter (here *R*_SR,1_) required to reproduce a particular observable (here *E*_–1_^hp^). The resulting curves that relate *N*_1_ and *R*_SR,1_ are shown in Figure 7. The curves do depend on *C*_6_ and the associated *C*_8_ and so are shown for values of *C*_6_ that vary by up to ±1% from the theoretical value in ref (69). As described below, *N*_1_ will ultimately be chosen on physical grounds, and the inset of Figure 7 shows how the required value of *R*_SR,1_ depends on *C*_6_ for the choice *N*_1_ = 10.

**Figure 8:**
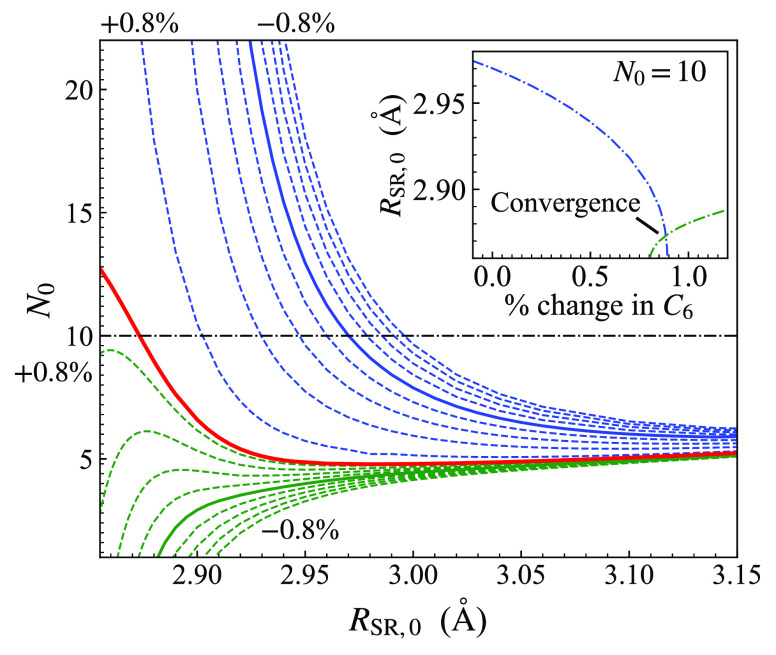
Relationship (green dashed lines) between the inverse power *N*_0_ and the short-range matching point *R*_SR,0_ required to reproduce the experimental binding energy *E*_–1_^ha^ of the least-bound state of NaCs in the ha channel. The relationship is given for various values of the dispersion coefficient *C*_6_, expressed as percentage differences from the theoretical value.^69^ The solid green curve shows the value used in ref (53). The blue lines show the analogous relationships required to reproduce the experimental position *B*_res_^aa^ of the s-wave resonance in the aa channel of Na + Cs. The solid red line is for the values of *C*_6_ required to reproduce *E*_–1_^ha^ and *B*_res_^aa^ simultaneously. The inset shows the dependence of *R*_SR,1_ on *C*_6_ required to fit each observable for the choice *N*_1_ = *N*_0_ = 10.

Once values are chosen for *C*_6_, *C*_8_, *N*_1_, and *R*_SR,1_, the triplet curve is fully defined. The same procedure may then be applied to vary the short-range part of the singlet curve to reproduce *E*_–1_^ha^. Because this state has multiple components as shown in Figure 3, this requires coupled-channel bound-state calculations, but it is nevertheless conceptually similar. The resulting relationship between *R*_SR,0_ and *N*_0_ is shown by the green lines in Figure 8, again for a range of values of *C*_6_.

We initially carried out this procedure with the dispersion coefficient *C*_6_ of ref (69), as used in ref (53). This produced the relationship between *R*_SR,1_ and *N*_1_ shown by the solid brown line in Figure 7 and between *R*_SR,0_ and *N*_0_ shown by the solid green line in Figure 8. It may be seen that, for the original value of *C*_6_, there is no value of *R*_SR,0_ that fits *E*_–1_^ha^ for *N*_0_ ≳ 5. Furthermore, the resulting potential curves fail to reproduce *B*_res_^aa^, the position of the resonance near 864 G in the aa channel; they place it near 873 G. This is because they place the zero-field binding energy of the state that causes this resonance significantly too deep, about 2470 MHz below the (*f*_Na_ = 2, *f*_Cs_ = 3) thresholds that support it. As seen in Figure 5, this is still a long-range state whose binding energy is controlled by the singlet and triplet scattering lengths and the dispersion coefficients. However, its wave function does not extend as far to long range as the least-bound states in Figure 3, so its binding energy is more sensitive to the dispersion coefficients than theirs. Because the relationship between *C*_6_ and *C*_8_ is determined by the binding energy of the absolute ground state and the singlet and triplet scattering lengths are determined by *E*_–1_^ha^ and *E*_–1_^hp^, the only way to adjust *B*_res_^aa^ is by varying *C*_6_ and *C*_8_.

We therefore repeat the calculation of the relationship between *R*_SR,0_ and *N*_0_, but by fitting to *B*_res_^aa^ instead of *E*_–1_^ha^. This produces the blue lines in Figure 8, again for a range of values of *C*_6_. It may be seen that the lines fitted to *B*_res_^aa^ and to *E*_–1_^ha^ are incompatible unless *C*_6_ is increased from its original value by approximately 0.9%. The inset of Figure 8 shows the values of *R*_SR,0_ obtained from each of the two fits for the choice *N*_0_ = *N*_1_ = 10. The requirement to fit both quantities produces a single value of *C*_6_ (and the corresponding *C*_8_ as required to reproduce *E*_00_ as above).

These results led us to an iterative procedure for fitting the experimental observable. We (i) choose values for *N*_0_, *N*_1_, and *C*_6_; (ii) vary *C*_8_ to fit *E*_00_; (iii) vary *R*_SR,1_ to fit *E*_–1_^hp^; (iv) vary *R*_SR,0_ to fit *E*_–1_^ha^; and (v) evaluate *B*_res_^aa^, adjust *C*_6_, and return to (ii). We repeat this cycle until convergence is achieved. This can be done for any reasonable values of *N*_0_ and *N*_1_, with results shown by the red line in Figure 7 and by the red line in Figure 8 for the choice *N*_1_ = 10. Any potential along these lines reproduces the four observables *E*_00_, *E*_–1_^hp^, *E*_–1_^ha^, and *B*_res_^aa^, and they differ very little in their predictions for other observable quantities. For our final interaction potential, we choose *N*_0_ = *N*_1_ = 10 to avoid the very soft repulsive wall of the triplet curve in ref (53).

It would have been possible to obtain the same final potential by a “blind” minimization procedure, but it conveys important insights to understand the interplay between parameters and the lines in parameter space that are capable of fitting each observable.

The parameters that differ from those in ref (53) are given in Table 1, together with the resulting singlet and triplet scattering lengths. Compared to ref (53), *R*_SR,0_ and *R*_SR,1_ have changed by 0.03 and −0.0072 Å, respectively; *N*_*S*_ has been fixed at a more physically reasonable value of 10 for both states, compared to its original value of 3; *C*_6_ has increased by 0.9%; in atomic units it is 3257(1)*E*_h_*a*_0_^6^, compared with 3227(18)*E*_h_*a*_0_^6^ from ref (69); and *C*_8_ has decreased by 3% from the fitted value of ref (53), but our fitted value corresponds to *C*_8_ = 3.568(4) × 10^5^*E*_h_*a*_0_^8^, which is closer to the theoretical value of *C*_8_ = 3.62(12) × 10^5^*E*_h_*a*_0_^8^ from ref (74) and well within its uncertainty.

**Table 1:** Parameters of the Fitted Interaction Potential, Including the Resulting Singlet and Triplet Scattering Lengths^a^

	singlet	triplet
*R*_SR,*S*_ (Å)	2.873240(6000)	4.772797(1600)
*N*_*S*_ (Å)	10	10
*A*_SR,*S*_/*hc* (cm^–1^)	–3798.0168	–420.536
*B*_SR,*S*_/*hc* (cm^–1^ Å^10^)	1.30971 × 10^8^	2.56041 × 10^9^
*a*_0,*S*_/*hc* (cm^–1^)	–4954.229 485	–217.146766
*C*_6_/*hc* (10^7^ cm^–1^ Å^6^)	1.568975(400)	
*C*_8_/*hc* (10^8^ cm^–1^ Å^8^)	4.815171(5000)	
*a*_s_ or *a*_t_ (*a*_0_)	433.05(65)	30.55(22)

a Only quantities that are different from those in ref (53) are listed. The derived parameters *A*_SR_, *B*_SR_, and *a*_0,*S*_, which arise from the continuity constraints applied to *V*(*R*) and *V*′(*R*), are included for convenience in evaluating the potential curves. The rounded values of *A*_SR_ correspond to the rounded values of *B*_SR_ and differ slightly from the values obtained with the exact *B*_SR_.

**Figure 9:**
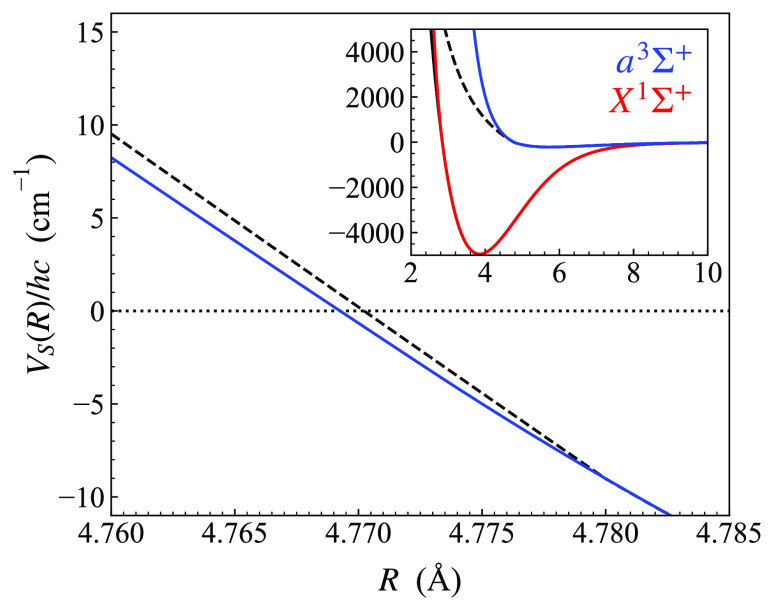
Comparison of the short-range region of the triplet curve of the present work (blue) with that of ref (53) (dashed black). The derivative discontinuity in the potential curve of ref is clearly visible.^53^ The inset shows the complete potential wells and the extrapolations onto the repulsive wall, including the singlet curve (red for the present work).

Key differences between our potential curves and those in ref (53) are shown in Figure 9. The derivative discontinuity in the triplet potential of ref (53) is clearly visible at 4.78 Å. The present triplet potential continues smoothly through *R*_SR,1_ and so has a zero-energy turning point at slightly shorter range, 4.7693 Å, compared to 4.7702 Å for the potential of ref (53). The effect of the larger values of *N*_0_ and *N*_1_ is seen in the steeper short-range repulsive walls shown in the inset.

#### 3.2.1 Uncertainties in Fitted Parameters

The interaction potential determined here is obtained by fitting four potential parameters to four experimental quantities. The 4-parameter space is actually a subspace of a much larger space, of approximately 50 parameters, that were fitted to FT spectra in ref (53). Reference (53) itself gave no uncertainties for the fitted parameters or estimates of the correlations between them. It is therefore not appropriate or practical to use error estimates based on deviations between observed and calculated properties. We can nevertheless make estimates of errors based on the derivatives of the calculated observables with respect to potential parameters, as described in Appendix C, and these are included in Table 1.

### 3.3 Predictions of the Fitted Potential

#### 3.3.1 Scattering Lengths

The singlet and triplet scattering lengths given in Table 1 are within the uncertainties of those obtained by Hood et al.,^46^*a*_s_ = 428(9)*a*_0_ and *a*_t_ = 30.4(6)*a*_0_. Their value of *a*_t_ was obtained from *E*_–1_^hp^, so it is of similar accuracy to ours, though ours is shifted slightly because we have determined improved values of the dispersion coefficients. Their value of *a*_s_ was obtained by combining *a*_t_ with measurements of interaction shifts, as described above. Our value of *a*_s_ is considerably more precise, both because of the greater precision of *E*_–1_^ha^ compared to the interaction shifts and because of the use of full coupled-channel calculations.

Hood et al. also gave the scattering length for the ha channel as −693*a*_0_, without an error estimate. This quantity is important because the large negative value enhances the intensity of photoassociation transitions originating from atoms in the ha state.^47^ Our interaction potential gives an even larger negative value of −860(2)*a*_0_. The value of ref (46) arose fairly directly from their measurements of interaction shifts, which are dominated by the ha channel. Our value is principally based on the more reliable and precise measurement of *E*_–1_^ha^, so it is expected to be more accurate.

In recent work, Warner et al.^75^ have created overlapping Bose–Einstein condensates of Na and Cs and measured the scattering length for the aa channel to be 18(4)*a*_0_ at *B* = 23 G and 29(4)*a*_0_ at *B* = 894 G. Our fitted interaction potential gives 14*a*_0_ at 23 G and 30*a*_0_ at 894 G, in good agreement with the measurements.

#### 3.3.2 Bound States with *L* = 0

**Figure 10:**
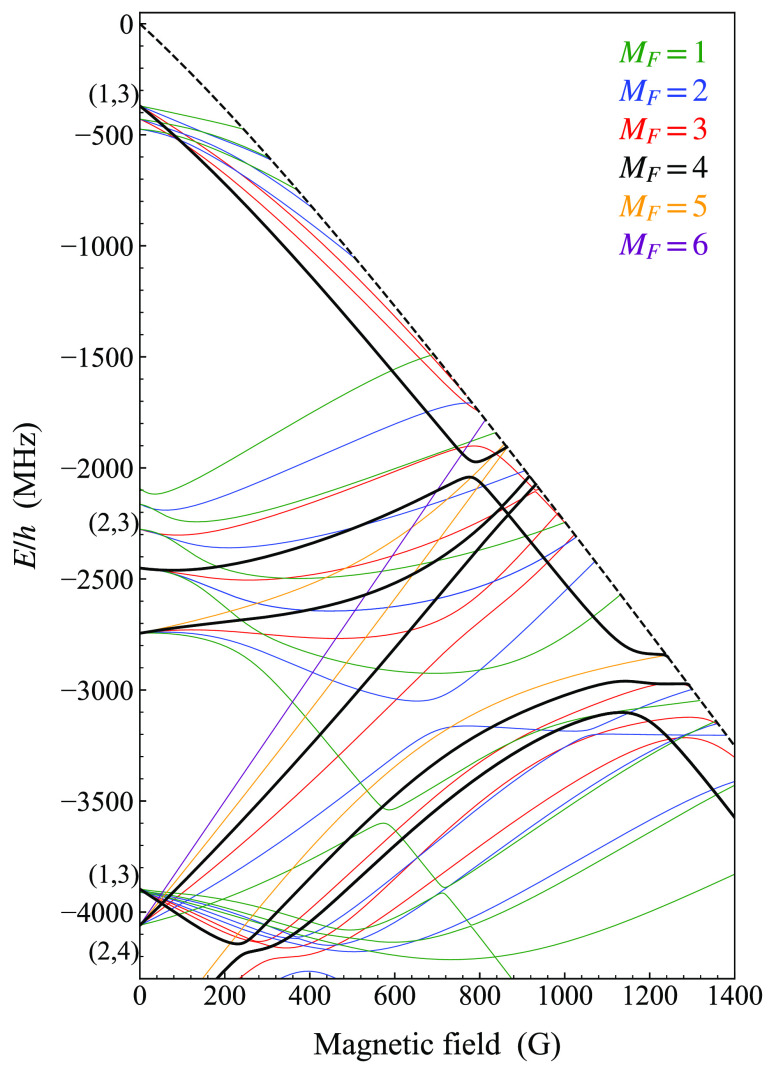
Weakly bound states of NaCs with *L* = 0 below the aa threshold as a function of magnetic field. The aa threshold is shown as a dashed black line. States with *M*_*F*_ = 4 that can cause s-wave Feshbach resonances are shown as solid black lines; other values of *M*_*F*_ are color-coded as shown in the legend. Only states with *M*_*F*_ from 1 to 6 are shown. The zero of energy is the threshold energy at zero field, which lies 6278.1 MHz below the hyperfine centroid.

Figure 10 shows the energies of bound states of NaCs below the lowest (aa) threshold as a function of magnetic field. All states with *M*_*F*_ between 1 and 6 are included (but not states with *M*_*F*_ from −6 to 0). The calculation uses a basis set with *L*_max_ = 0, so only states with *L* = 0 are shown. At zero field, the states can be grouped according to their hyperfine characters. The uppermost group, with zero-field binding energies from 350 to 500 MHz, are *n* = −1 states with character (*f*_Na_, *f*_Cs_) = (1, 3). The next group, from 2000 to 2800 MHz, are *n* = −2 states with character (2, 3). The group near 3900 MHz has character (1, 3) but with *n* = −2. Finally, the deepest group shown, which starts slightly deeper than 4000 MHz and extends off the bottom of the plot, is made up of *n* = −3 states with character (2, 4).

For each group, *f*_Na_ couples to *f*_Cs_ to give a resultant *F*, which is a good quantum number at zero field. The allowed values of *F* run from *f*_Cs_ – *f*_Na_ to *f*_Cs_ + *f*_Na_ in steps of 1. In a magnetic field, each state splits into components with different *M*_*F*_ values (though not all possible values of *M*_*F*_ are shown). The value of *F* for a zero-field state can therefore be inferred from the largest *M*_*F*_ present. *M*_*F*_ is a good quantum number when *L*_max_ = 0, but at moderate fields (between 30 and 500 G), states of the same *M*_*F*_ but different *F* approach one another and mix; above these fields, *m*_*f*,Na_ and *m*_*f*,Ca_ are better quantum numbers than *F*.

#### 3.3.3 Resonances in s-Wave Scattering

It is important to distinguish between *L*_in_ for the incoming wave and *L* for a bound state. The widest resonances in s-wave scattering (*L*_in_ = 0) are due to s-wave bound states (with *L* = 0) and are referred to as s-wave resonances. Because *M*_tot_ = *M*_*F*_ + *M*_*L*_ is conserved and is 4 for an incoming s wave at the aa threshold, bound states with *L* = 0 can cause resonances at this threshold only if they have *M*_*F*_ = 4. These states are shown as solid black lines in Figure 10.

Bound states with even *L* > 0 can also cause Feshbach resonances in s-wave scattering, which are usually narrower. The widest of these are d-wave resonances due to d-wave states (with *L* = 2). In this case, *M*_*L*_ can take values from −2 to 2, so d-wave states with *M*_*F*_ = 2 to 6 can have *M*_tot_ = 4 and cause resonances in s-wave scattering at the aa threshold.

**Figure 11:**
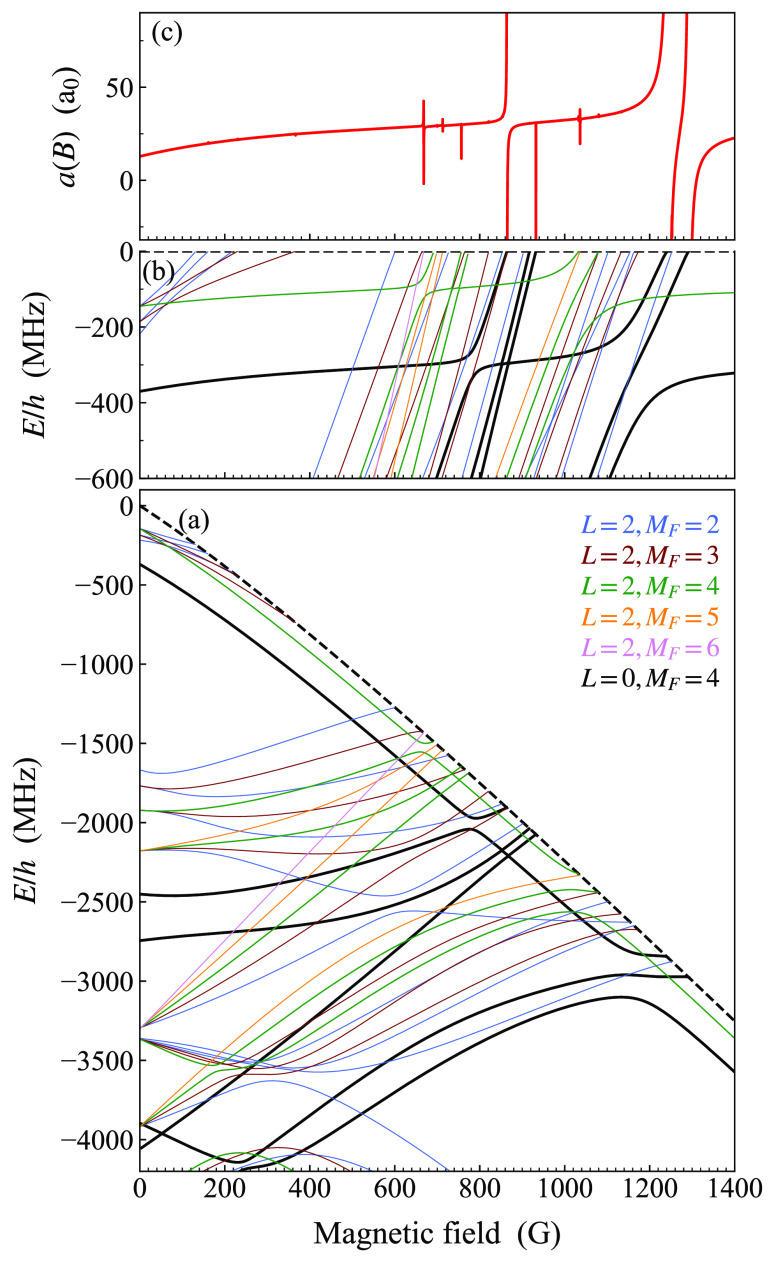
(a) Weakly bound states of NaCs with *M*_tot_ = 4 and *L* = 0 or 2 below the aa threshold as a function of magnetic field. The aa threshold is shown as a dashed black line. States with *L* = 0 and *M*_*F*_ = 4 that can cause s-wave Feshbach resonances are shown as solid black lines; states with *L* = 2 that can cause d-wave resonances are color-coded according to *M*_*F*_ as shown in the legend. The zero of energy is the threshold energy at zero field. (b) Expanded view of (a), with energies shown as binding energies with respect to the aa threshold. (c) s-wave scattering length at the aa threshold, showing resonances where bound states cross the threshold. Some of the resonances that exist are too narrow to see on the 0.2 G grid used for the calculation of the scattering length.

Figure 11(a) shows all states with *M*_tot_ = 4 that lie close to the aa threshold, as a function of magnetic field. This calculation uses a basis set with *L*_max_ = 2, so it includes states with both *L* = 0 and 2. States with *L* = 0 and *M*_*F*_ = 4 are again shown in black, whereas states with *L* = 2 are color-coded according to *M*_*F*_. To allow this labeling, the small couplings off-diagonal in *M*_*F*_ are neglected in the bound-state calculations (but not in the corresponding scattering calculations). The pattern of zero-field states for each hyperfine group is similar in structure to Figure 10, but the states with *L* = 2 are shifted upward by a rotational energy. Figure 11(b) shows an expanded view of the bound states, plotted as energies below the aa threshold, and Figure 11(c) shows the resulting s-wave scattering length. A resonance occurs at every field where a state with *M*_tot_ = 4 crosses the threshold, but some of them are too narrow to be visible on the grid of magnetic fields used for Figure 11(c). Nevertheless, all of them can be characterized in scattering calculations, using the methods of ref (60), to give values for *B*_res_, Δ, and *a*_bg_ from eq 5.

Table 2 gives the parameters of all s-wave and d-wave resonances with Δ > 10^–4^ G, together with quantum numbers for the states that cause them. It may be noted that the s-wave resonance near 864 G, which appeared at 864.11 G in a calculation with *L*_max_ = 0, is shifted to 864.13 G in the calculation with *L*_max_ = 2. This demonstrates the small effect of basis functions with *L* = 2 on s-wave properties and justifies the use of *L*_max_ = 0 in fitting.

**Table 2:** Feshbach Resonances with Widths Greater Than 10^–4^ G in s-Wave and p-Wave Scattering at the aa Threshold^a^

resonances in s-wave scattering (34 total)				
*B*_res_ (G)	Δ (G)	*a*_bg_ (*a*_0_)	*L*	*M*_*F*_
161.23	0.0007	19.8	2	2
218.30	0.0002	21.6	2	2
230.24	0.0007	21.9	2	3
366.36	0.0010	24.8	2	3
668.14	0.066	28.9	2	6
699.69	0.0012	29.2	2	5
712.89	0.011	29.4	2	5
756.80	0.0016	29.9	2	4
773.90	0.0002	30.2	2	4
853.50	0.0008	34.2	2	2
864.13	1.27	30.7	0	4
864.42	–0.0001	–105	2	3
917.07	0.0003	30.6	0	4
932.20	0.0003	30.9	0	4
1032.90	0.0035	33.2	2	4
1036.15	0.022	33.0	2	5
1080.00	0.001	34.3	2	3
1133.52	0.0005	36.9	2	3
1243.02	14.4	40.2	0	4
1252.53	–0.026	–22.6	2	2
1292.57	17.7	20.5	0	4

resonances in p-wave scattering (17 total)				
*B*_res_ (G)	Δ (G)	*v*_bg_ (10^7^ *a*_0_^3^)	*L*	*M*_*F*_
805.41	0.021	–1.50	1	4
806.80	0.0083	–1.53	1	5
1173.87	0.42	–1.50	1	4
1216.83	0.0067	–1.47	1	3
1222.78	0.21	–1.53	1	4

a The p-wave calculations are for *M*_tot_ = 4 only.

Zhang et al.^47^ observed a weak d-wave Feshbach resonance at 864.5 G on the shoulder of the s-wave resonance at 864.11 G. The bound state responsible for this is visible in Figure 11(a) and crosses the threshold at 864.42 G, causing a resonance of width Δ = −10^–4^ G. It is an impressive demonstration of the quality of our interaction potential that it can reproduce the position of this resonance to within 0.1 G and identify the bound state responsible: it is a state with *L* = 2, *M*_*F*_ = 3 (brown in Figure 11) involving a pair of states originating from (*f*_Na_, *f*_Cs_, *F*) = (2, 3, 5) and (2, 4, 6) that experience an avoided crossing at around 700 G.

#### 3.3.4 Resonances in p-Wave Scattering

Resonances can also occur in p-wave scattering (*L*_in_ = 1) due to either p-wave states (with *L* = 1) or states with higher odd *L*. In the gas phase, such resonances are usually observed only at relatively high temperatures (several μK), but in optical tweezers it is possible to enhance them selectively by promoting one atom to a motionally excited state. Zhang et al.^47^ observed a group of p-wave resonances at around 807 G for Na + Cs, with complicated structure, and used them to produce a single p-wave molecule in the tweezer.

**Figure 12:**
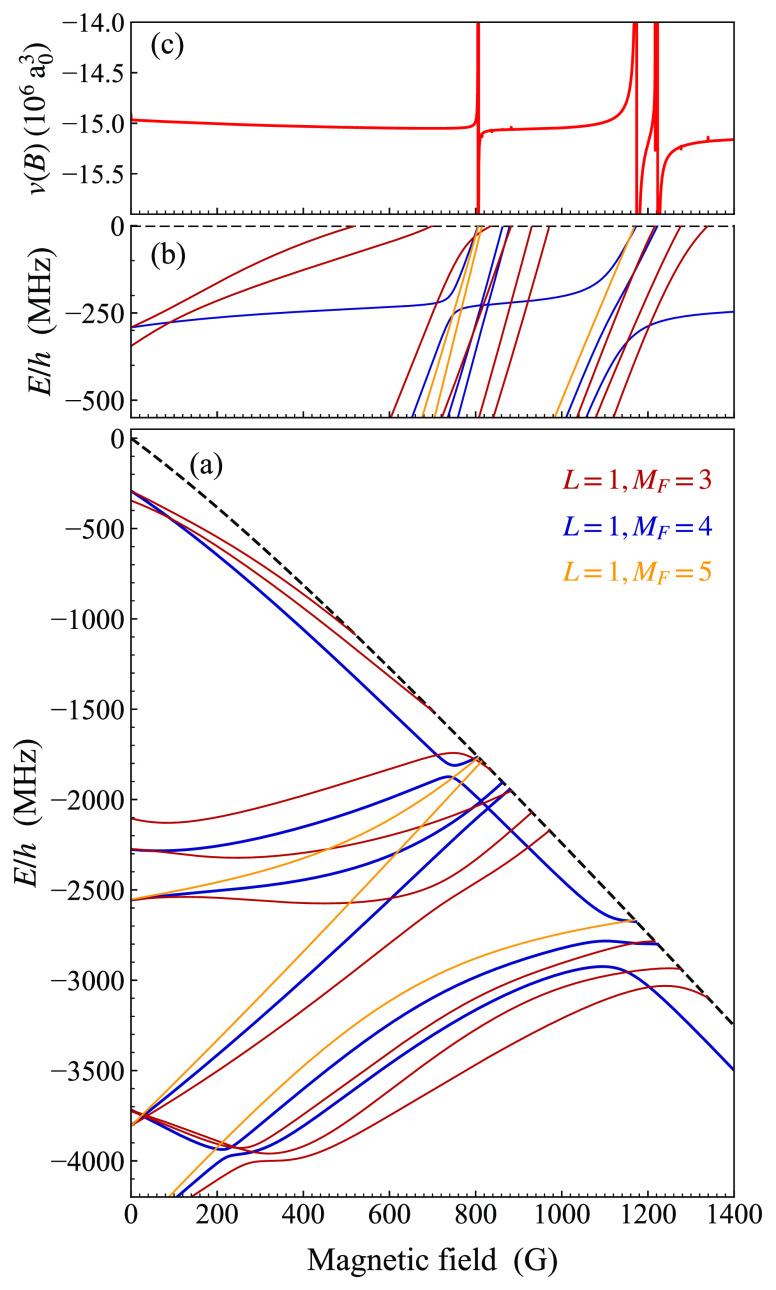
(a) Weakly bound p-wave states of NaCs, with *M*_tot_ = 4 and *L* = 1, below the aa threshold as a function of magnetic field. The aa threshold is shown as a dashed black line. Only states with *M*_tot_ = 4 are shown. The states are color-coded according to *M*_*F*_ as shown in the legend. The zero of energy is the threshold energy at zero field. (b) Expanded view of (a), with energies shown as binding energies with respect to the aa threshold. (c) p-wave scattering volume at the aa threshold, calculated at a collision energy of 2 μK × *k*_B_. Some of the resonances that exist are too narrow to see on the 0.2 G grid used for the calculation of the scattering volume.

For p-wave scattering, *M*_*L*,in_ can be −1, 0, or −1 and *M*_tot_ = *M*_*F*,in_ + *M*_*L*,in_. Thus, even at the aa threshold, *M*_tot_ can be 3, 4, or 5. If the resonant state has *L* = 1, *M*_*L*_ can be −1, 0, or −1 too. For each of the three values of *M*_tot_, p-wave resonances arise from bound states with *M*_*F*_ = *M*_tot_ and *M*_tot_ ± 1. Figure 12(c) shows the p-wave bound states below the aa threshold and the corresponding scattering volume *v*, but only for the case *M*_tot_ = 4. The bound states show considerable similarities to the s-wave and p-wave ones in Figures 10 and 11. Figure 12(c) shows that s-wave and p-wave states share several similarities, but with shifts due to the different rotational energy in each case. The positions, widths, and assignments of the widest resulting resonances are given in Table 2, but it must be remembered that this is for only one of the three possible values of *M*_tot_ for p-wave scattering at the aa threshold. Figure 12 and Table 2 show that the group of resonances observed^47^ near 807 G are mainly the p-wave analogs of the s-wave resonance near 864 G.

#### 3.3.5 Resonance in the cg Channel

As described above, Hood et al.^46^ measured the position of an inelastic loss feature in the cg channel at 652.1(4) G. Our fitted potential produces a resonance at 654.3 G. However, its width is Δ = 43 G, so the difference between the resonance position and the observed loss peak is only 5% of the width. The calculated background scattering length is −41*a*_0_.

## 4 Conclusions

We have used measurements on ultracold scattering and spectroscopy in optical tweezers,^43,45−48^ combined with previous work using Fourier transform spectroscopy,^53^ to determine improved potential curves for the singlet and triplet states of NaCs. We have used coupled-channel calculations based on these curves to characterize the weakly bound states involved and to make predictions for additional bound states and Feshbach resonances.

Each measurement of a spectroscopic transition or resonance position is sensitive to the properties of one or two specific bound states of the molecule. These properties are in turn sensitive to particular features of the interaction potentials. Our work has produced important insights into these relationships and the ways that combinations of measurements can be used to determine features of the potential curves.

For NaCs, as for many other diatomic molecules, the mid-range parts of the potential curves had previously been accurately determined from spectroscopy at relatively high temperatures. For NaCs, this mid-range part extends from just outside the inner turning point at the dissociation energy to 10.2 Å and is expressed as a power-series expansion for each of the singlet and triplet curves.^53^ Our approach is to change the mid-range part by as little as possible to retain its ability to fit the higher-temperature spectra. We thus retain the mid-range expansion unchanged and adjust only the extrapolations to long and short range. This gives sufficient flexibility to reproduce the ultracold observables.

The binding energy of the least-bound (uppermost) state in a particular scattering channel, *E*_–1_, is closely related to the scattering length *a* for that channel. The relationship between *E*_–1_ and *a* depends on the dispersion coefficients for the long-range interaction, particularly *C*_6_, but only weakly. Because the dispersion coefficients are often known fairly accurately from independent theory,^69^*E*_–1_ is a good surrogate for *a*. If it can be measured for two channels that represent significantly different mixtures of singlet and triplet states, the singlet and triplet scattering lengths *a*_s_ and *a*_t_ can be disentangled. This is the case for NaCs, where *E*_–1_ has been measured both for a spin-stretched channel that is pure triplet in character^45,46^ and for the ha channel,^48^ which has about 50% singlet character. Because the mid-range part of the potential is held fixed to reproduce the higher-temperature spectra and the dispersion coefficients have only limited influence, the two values of *E*_–1_ determine the short-range parts of the singlet and triplet curves.

Magnetic Feshbach resonances exist where a weakly bound molecular state crosses a scattering threshold as a function of magnetic field. These states are often supported by thresholds in which one or both atoms are in excited hyperfine states. States that cause resonances at the lowest threshold are thus often bound by considerably more than the least-bound state. In NaCs, the state that causes the resonance observed in the lowest channel^47^ is bound by more than 4 GHz with respect to the threshold that mostly supports it. Because of this, it is much more sensitive to the dispersion coefficients than the least-bound states. The requirement to reproduce this resonance position as well as the least-bound states places a strong constraint on the dispersion coefficients, particularly *C*_6_.

In potential curves from higher-temperature spectroscopy, the dissociation energy (and thus the absolute binding energies of all of the deeply bound states) is usually obtained from extrapolation rather than measured directly. However, Raman transfer of ultracold molecules to a deeply bound state provides a direct measurement of its absolute binding energy. If the mid-range part of the potential is held fixed to reproduce the higher-temperature spectra, this provides a second (and different) constraint on the dispersion coefficients. Satisfying this along with the constraint from the resonance position allows *C*_6_ and *C*_8_ to be disentangled.

There is an important general insight here. The spectroscopy of ultracold molecules often provides measurements of the energies of the least-bound molecular states supported by one or more thresholds. Measurements of tunable Feshbach resonances are often sensitive to somewhat deeper states, with binding energies in the GHz range. When such measurements are combined, they can provide very precise values for dispersion coefficients. The same principle applies when different Feshbach resonances provide implicit information on two or more states with substantially different binding energies with respect to the thresholds that support them.

For NaCs, we find that the different ultracold observables can be fitted simultaneously only if *C*_6_ is increased by about 0.9% from the theoretical value. Our fitted value corresponds to 3256(1)*E*_h_*a*_0_^6^, compared to 3227(18)*E*_h_*a*_0_^6^ from ref (69). Our fitted value *C*_8_ = (3.568(4) × 10^5^)*E*_h_*a*_0_^8^ is well within the error bounds of the value of ref (74).

Accurately fitted interaction potentials are key to progress in ultracold scattering and spectroscopy. They provide predictions of new experimental observables, which are often crucial in designing experiments and locating new spectroscopic lines. They also provide calculated scattering lengths, as a function of magnetic field, which are unavailable from other sources. These are often crucial in experiments that need precise control of the scattering length, such as those exploring Efimov physics or quantum phase behavior.

## Appendix A: Magnetic Dipole Interaction and Second-Order Spin–Orbit Coupling

At long range, the coupling *V̂*^d^(*R*) of eq 3 has a simple magnetic dipole–dipole form that varies as 1/*R*^3^.^55,76^ However, for heavy atoms such as Cs, second-order spin–orbit coupling provides an additional contribution that has the same tensor form as the dipole–dipole term and dominates at short range.^77,78^ In the present work, *V̂*^d^(*R*) is written as

15

where *e⃗*_*R*_ is a unit vector along the internuclear axis and λ is an *R*-dependent coupling constant. This term couples the electron spins of Na and Cs atoms to the molecular axis.

The second-order spin–orbit splitting is not known for NaCs. However, it contributes only when *L*_max_ > 0 and makes only very small contributions for s-wave states and resonances due to them. We model it here using the functional form used for RbCs,^79^

16

where *E*_h_ is the Hartree energy and α ≈ 1/137 is the atomic fine-structure constant. To account for the smaller size of Na compared to Rb, we adjust the values of *A*_2SO_^short^ and *A*_2SO_^short^ for RbCs to shift the second-order spin–orbit contribution to short range by 0.757*a*_0_. This gives parameters *A*_2SO_^short^ = −27.8, *A*_2SO_^long^ = −0.027, β_2SO_^short^ = 0.80, and β_2SO_^long^ = 0.28 for NaCs. Future experiments may allow the determination of these parameters, but changing them would have little effect on the singlet and triplet curves obtained here (though it might have a significant effect on the widths of predicted d-wave resonances).

## Appendix B: Coupled-Channel Methods

We expand the total wave function of the molecule or colliding pair of atoms in a coupled-channel representation,

17

Here, ξ represents all coordinates of the pair except the internuclear distance *R*. The functions Φ_*j*_(ξ) form a complete orthonormal basis set for motion in the coordinates ξ, and the factor *R*^–1^ serves to simplify the action of the radial kinetic energy operator. The component of the wave function in each channel *j* is described by ψ_*j*_(*R*), and these are the functions shown in Figures 3 and 5. The expansion (eq 17) is substituted into the total Schrödinger equation, and the result is projected onto a basis function Φ_*i*_(ξ). The resulting coupled differential equations for the functions ψ_*i*_(*R*) are

18

where δ_*ij*_ is the Kronecker delta, , *E* is the total energy, and

19

The different equations are coupled by the off-diagonal terms *W*_*ij*_(*R*) with *i* ≠ *j*.

The coupled equations may be expressed in matrix notation,

20

If there are *N* basis functions included in the expansion (eq 17), **ψ**(*R*) is a column vector of order *N* with elements ψ_*j*_(*R*), **I** is the *N* × *N* unit matrix, and **W**(*R*) is an *N* × *N* interaction matrix with elements *W*_*ij*_(*R*).

In general, there are *N* linearly independent solution vectors **ψ**(*R*) that satisfy the Schrödinger equation subject to the boundary condition that **ψ**(*R*) → 0 in the classically forbidden region at short range. These *N* column vectors form a wave function matrix **Ψ**(*R*).

## Appendix C: Uncertainties in Fitted Parameters

Our objective is to fit a set of *M* parameters *p*_*j*_, collectively represented by the vector **p**, to a set of *N* observables *y*_*i*_^obs^. We minimize the weighted sum of squares of residuals,

21

where *u*_*i*_ is an uncertainty for observable *i*. In standard least-squares methods, with *N* ≫ *M*, the common uncertainty of the measurements is usually estimated statistically from the minimum value of χ^2^ achieved in the fit, generally with a denominator *N* – *M*. In the present work, *N* = *M* = 4, so this is not possible. Instead, we choose the values *u*_*i*_ as the experimental uncertainties.

To estimate uncertainties in the fitted parameters, we follow the usual procedures for nonlinear least-squares fitting. At the final values of the parameters, we calculate a 4 × 4 Jacobian matrix **J** with elements . We scale this by the chosen uncertainties to define the matrix **A** with elements *A*_*ij*_ = *J*_*ij*_/*u*_*i*_ and the Hessian matrix **H** = **A**^*T*^**A**; the elements of the latter are half the second partial derivatives of χ^2^ with respect to potential parameters. We choose uncertainties in the parameters defined by a contour at χ^2^ = 1. The variance–covariance matrix is then **Θ** = **H**^–1^. The resulting correlated uncertainties Θ_*jj*_^1/2^ are ±0.006 Å in *R*_SR,0_, ±0.0016 Å in *R*_SR,1_, ±4 × 10^3^ cm^–1^ Å^6^ in *C*_6_, and ±5 × 10^5^ cm^–1^ Å^8^ in *C*_8_. The correlation matrix has elements ; all elements have a magnitude below 0.6 except that between *R*_SR,0_ and *R*_SR,1_, which is −0.995.

It should be noted that these uncertainties do not take account of model dependence due to fixing the parameters of the mid-range potential. These are hard to estimate in a systematic way because ref (53) did not discuss uncertainties in the parameters or the correlations between them.

In correlated fits, it often is not sufficient to specify parameters to within their uncertainties. The sensitivity of calculated properties to the parameters depends on the Hessian matrix **H** rather than its inverse **Θ**.^80^ To reproduce the observables to within their uncertainties, each parameter must be specified to a precision of at least *H*_*jj*_^–1/2^, which may be much smaller than Θ_*jj*_^1/2^. The parameters in Table 1 are given to a precision based on these values.
